# *Ndrg2* promoter hypermethylation triggered by helicobacter pylori infection correlates with poor patients survival in human gastric carcinoma

**DOI:** 10.18632/oncotarget.3601

**Published:** 2015-03-15

**Authors:** Zhi-Qiang Ling, Ming-Hua Ge, Xiao-Xiao Lu, Jin Han, Yi-Chen Wu, Xiang Liu, Xin Zhu, Lian-Lian Hong

**Affiliations:** ^1^ Zhejiang Cancer Research Institute, Zhejiang Province Cancer Hospital, Zhejiang Cancer Center, Hangzhou, China; ^2^ Department of Tumor Surgery, Zhejiang Province Cancer Hospital, Zhejiang Cancer Center, Hangzhou, China

**Keywords:** gastric carcinoma, Helicobacter pylori (H. pylori) infection, DNMT3b, N-myc downstream regulated gene 2 (Ndrg2), promoter methylation

## Abstract

N-myc downstream regulated gene 2 (*Ndrg2*) is a candidate suppressor of cancer metastasis. We found that *Ndrg2* promoter was frequently hypermethylated in gastric cancer cell lines and in 292 gastric tumor tissues. This resulted in down-regulation of *Ndrg2* mRNA and protein. *Ndrg2* promoter methylation was associated with H. *pylori* infection and worse prognosis of gastric cancer patients, which is an independent prognostic factor for the disease-free survival (DFS). We found that H. *pylori* silenced *Ndrg2* by activating the NF-κB pathway and up-regulating DNMT3b, promoting gastric cancer progression. These findings uncover a previously unrecognized role for H. *pylori* infection in gastric cancer.

## INTRODUCTION

Gastric cancer (GC) is one of the most deadly diseases worldwide, mainly attributing to the high frequency of metastasis [1]. Being predominantly found in older patients with a long period of atrophic gastritis, GC is thought to result from a combination of environmental factors and the accumulation of genetic alterations [2-4]. The commonest cause of gastritis is the infection of *H*. *pylori*, which is the single most common risk factor of gastric cancer [5, 6]. *H. pylori* has been classified as a class I carcinogen by the WHO since 1994 [7, 8] and its causal role in GC has been extensively studied in animal models [9]. However, the role of *H*. *pylori* infection in the tumorigenesis and tumor progression of gastric cancer is largely elusive.

Human N-Myc downstream-regulated gene (*Ndrg)* family consists of *Ndrg1*, *Ndrg2*, *Ndrg3*, and *Ndrg4* which locate on chromosomes 8q24.3, 14q11.2, 20q11.21-11.23, and 16q21-q22.1, respectively [10]. Functions of Ndrg family have been associated with cell proliferation, differentiation, apoptosis, stress responses, and cell migration/metastasis [11-13]. Down-regulation of *Ndrg1* and *Ndrg2* have been reported in various cancer tissues, and have been regarded as tumor suppressors and/or metastasis suppressors [14, 15]. *Ndrg2* contains a CpG island in its promoter region and DNA hypermethylation has been reported in pancreatic cancer [16], glioblastoma [17, 18], adrenocortical carcinomas [19], breast cancer [20, 21], colorectal cancer [22, 23], oral squamous-cell carcinoma [24], meningioma [25], liver [26], and gastric cancer [27, 28]. The methylation of *Ndrg2* was found in 54.0 % (47/87) of primary gastric cancer specimens and related to gastric cancer progression [27]. However, till now, nothing has been reported about the relationship between *Ndrg2* methylation and *H. pylori* infection in gastric mucosa epithelial cells.

In this study, we investigated the expression level and methylation status of *Ndrg2* in 292 gastric cancer and matched noncancerous tissues, and we evaluated the clinical significance of abnormal *Ndrg2* methylation in gastric cancer. We also evaluated the correlation between *Ndrg2* methylation and *H. pylori* infection in gastric cancer. Finally, we explored the possible mechanism of *H. pylori* infection in mediating *Ndrg2* promoter methylation *in vitro*. Our results suggested that *H. pylori* may function as an initiator in the progression of gastric cancer by regulating DNA methyltransferase 3b (DNMT3b).

## RESULTS

### Ndrg2 is significantly down-regulated in human gastric cancer cells and primary gastric cancer tissues

**Figure 1:**
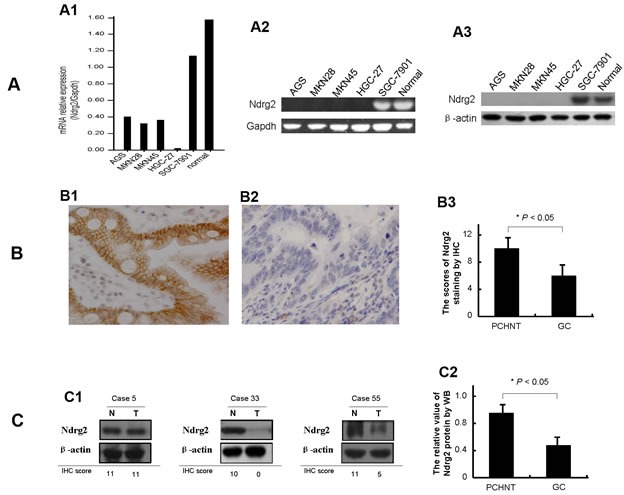
Expression of Ndrg2 is significantly reduced in gastric cancer cells The relative level of *Ndrg2* transcript in gastric cancer cell lines in comparison with normal gastric mucosa epithelial tissues from patients undergoing gastroscope examination is shown in A1. *Ndrg2* mRNA level was determined by RT-PCR and adjusted for Gapdh. The tumor/PCHNT ratio < 0.5 is considered as low expression and > 2 to be considered as high expression. The mRNA level of *Ndrg2* in gastric cancer cell lines as determined by 2.0% agarose gel electrophoresis is shown in A2. The protein level of Ndrg2 in gastric cancer cell lines as determined by western blot (WB) is shown in A3. β-actin was internal reference. The Ndrg2 protein results by western blot compared with the *Ndrg2* mRNA results by RT-PCR have the better consistency. Representative immunohistochemical (IHC) images of Ndrg2 expression are shown for normal gastric tissues (B1) and cancer tissues (B2). ×200 for all images. The protein level of Ndrg2 in 292 primary gastric cancer tissues as determined by IHC is shown in B3. IHC score of 0-4 is considered to be no to low expression and 5-12 considered to be normal to high expression. The level of Ndrg2 expression in PCHNTs is higher than that in tumor tissues (*P*<0.05). The protein level of Ndrg2 in thirty specimens of gastric cancer and paired normal gastric epithelium tissues as determined by WB is shown in C. Representative WB images of Ndrg2 expression are shown in C1, and in comparison with IHC results. β-actin was internal control. Western blot analysis of Ndrg2 protein showed that the differences between tumor and PCHNT groups were significant (*P*<0.05) (C2).

To investigate the candidacy of *Ndrg2* as a suppressor in gastric progression, we initially characterized the expression status of *Ndrg2* transcript in five gastric cancer cell lines. *Ndrg2* mRNA expression is significant lower in 4 of 5 gastric cancer cell lines as compared to the average level of 40 normal gastric mucosas from healthy individuals examined (Figure 1 A1-A2); almost undetectable in HGC-27 cells and relative higher in SGC-7901 cells (Figure 1 A1-A2). Western blotting analysis validated the mRNA expression results, confirming that the Ndrg2 protein levels in the 4 cell lines are non-detectable (Figure 1 A3).

Next, we evaluated *Ndrg2* mRNA expression level in 292 primary gastric cancer tissues, as well as their corresponding para-cancerous histological normal tissue (PCHNT) specimens and 125 non-cancer volunteers. mRNA expression level (*Ndrg2* /*Gapdh*) in GCs (0.135–2.062, mean=0.749) is markedly reduced as compared to PCHNTs (0.248-1.972; mean=1.367, *P*<0.05), dysplasia (0.772-2.004; mean=1.755, *P*<0.05), chronic non-atrophic gastritis (0.786-2.042; mean=1.685, *P*<0.05), and healthy individuals (1.266-2.041; mean=1.59, *P*<0.05), respectively (Supplementary Figure 1). There was no significant difference in *Ndrg2* expression in PCHNTs or in dysplasia as compared to other non-cancer controls (all *P*>0.05).

To confirm the above mRNA results, we subsequently analyzed Ndrg2 protein expression in 292 GCs and their corresponding PCHNTs using immunohistochemical (IHC) assay. Ndrg2 protein was expressed in the cytoplasm and plasma membrane in histological normal gastric epithelial cells (PCHNTs) with a moderate to strong staining intensity (Figure 1 B1). However, gastric cancer tumor cells displayed significantly reduced Nrdg2 protein expression as compared to PCHNT (Figure 1 B2-B3). We also carried out western blot analyses to confirm the above IHC findings in 30 randomly selected matched primary PCHNT and tumor tissue samples. Some representative western blot results are shown in Figure 1 C1. The average bands intensity of Ndrg2 in gastric cancer is significantly lower than that in PCHNT (*P*<0.05, Figure 1 C2). The protein levels of Ndrg2 determined by IHC in the 292 GC samples correlated well with the mRNA level of *Ndrg2* detected by real-time RT-PCR (r=0.714, *P*=0.000) (Figure 3). We also analyzed the correlation between Ndrg2 IHC results with clinicopathological factors. The data shows that loss of Ndrg2 protein significantly correlated with *H. pylori* (HP) infection (*P*=0.000), venous invasion (*P*=0.011), lymphatic involvement (*P*=0.003), invasive depth (*P*=0.000), lymph node metastasis (*P*=0.000), distant metastasis (*P*=0.000) and clinical stage (*P*=0.000) (Table 1). Collectively, these data indicate that the expression of Ndrg2 is significantly reduced at mRNA and protein levels in GC cells, and loss of Ndrg2 expression may associate with tumor progression and metastasis.

**Table 1:** Correlation between *Ndrg2* mRNA, Ndrg2 protein, *Ndrg2* methylation and clinicopathologic parameters, respectively

Clinicopathological parameters	Number of cases	*Ndrg2* mRNA down-regulation (%)	P*	Ndrg2 protein down-regulation (%)	P*	*Ndrg2* methylation (%)	P*
Cases							
Tumor	292	103 (35.3)	3.500E-11	108 (37.0)	4.791E-15	192 (65.8%)	1.198E-41
PCHNT	292	35 (12.0)		28 (9.6)		33 (11.3)	
Gender							
Male	168	54 (32.1)	0.192	58 (34.5)	0.310	108 (64.3)	0.538
Female	124	49 (39.5)		50 (40.3)		84 (67.7)	
Age at diagnosis							
<60	109	38 (34.9)	0.910	39 (35.8)	0.742	67 (61.5)	0.234
≥60	183	65 (35.5)		69 (37.7)		125 (68.3)	
Tumor site							
Cardia	56	15 (26.8)	0.139	15 (26.8)	0.079	33 (58.9)	0.231
Body/Antrum	236	88 (37.3)		93 (39.4)		159 (67.4)	
Tissue type							
Diffuse	96	37 (38.5)	0.413	39 (40.6)	0.367	64 (66.7)	0.818
Intestinal	196	66 (33.7)		69 (35.2)		128 (65.3)	
H. pylori							
No	131	24 (18.3)	4.527E-08	23 (17.6)	5.527E-10	43 (32.8)	1.062E-26
Yes	161	79 (49.1)		85 (52.8)		149 (92.5)	
Tumor size							
<5cm	90	30 (33.3)	0.643	27 (30.0)	0.099	47 (52.2)	0.001
≥5cm	202	73 (36.1)		81 (40.1)		145 (71.8)	
Histological differentiation							
Well/Moderately	101	29 (28.7)	0.088	30 (29.7)	0.061	59 (58.4)	0.055
Poorly	191	74 (38.7)		78 (40.8)		133 (69.6)	
Venous invasion							
Negative	140	39 (27.9)	0.011	40 (28.6)	0.004	57 (40.7)	5.000E-18
Positive	152	64 (42.1)		68 (44.7)		135 (88.8)	
Lymphatic invasion							
Negative	116	29 (25.0)	0.003	28 (24.1)	2.224E-04	41 (35.3)	6.121E-19
Positive	176	74 (42.0)		80 (45.5)		151 (85.8)	
Invasive depth						
T1/T2	121	21 (17.4)	7.029E-08	17 (14.0)	8.530E-12	46 (38.0)	4.394E-17
T3/T4	171	82 (48.0)		91 (53.2)		146 (85.4)	
Lymph node metastasis							
Negative	104	23 (22.1)	4.652E-04	25 (24.0)	0.001	36 (34.6)	7.4452E-17
Positive	188	80 (42.6)		83 (44.1)		156 (83.0)	
Distant metastasis							
Negative	220	60 (27.3)	5.679E-07	64 (29.1)	1.034E-06	121 (55.0)	1.29801E-11
Positive	72	43 (59.7)		44 (61.1)		71 (98.6)	
TNM stage							
Stage I/II	84	2 (2.4)	7.695E-14	3 (3.6)	5.637E-14	7 (8.3)	1.94E-39
Stage III/IV	208	101 (48.6)		105 (50.5)		185 (88.9)	

Down: T/N < 0.5 in mRNA, IHC score ≤4.

M: methylated score ≥1.

*P*: Pearson Chi-Square Tests.

### Frequent DNA hypermethylation at the CpG sites in the *Ndrg2* promoter

**Figure 2:**
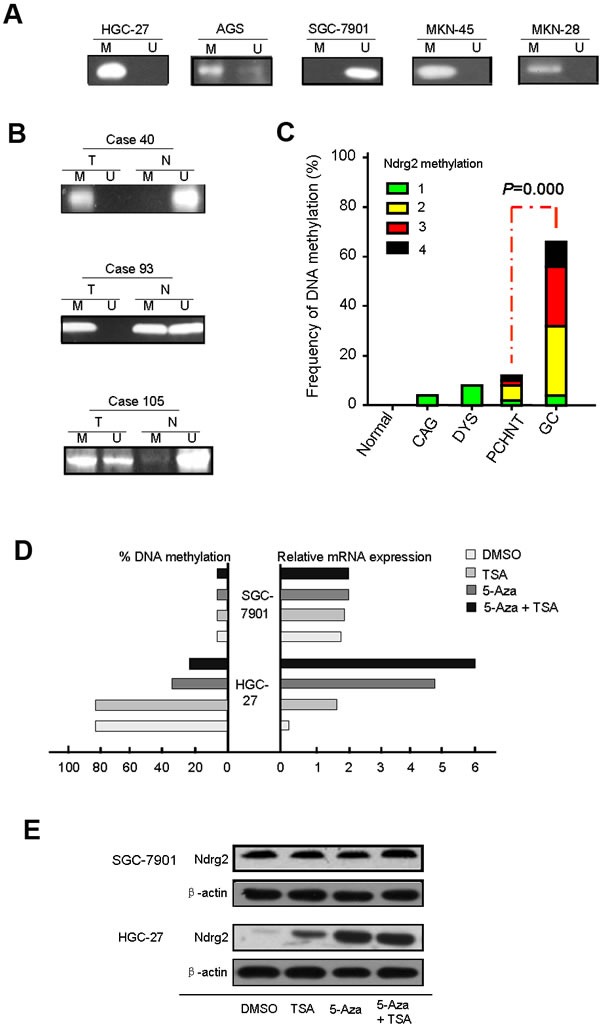
The analysis of aberrant DNA hypermethylation at the CpG sites in the Ndrg2 promoter The methylated level of *Ndrg2* in cell lines, primary gastric cancer tissues and paired PCHNTs after real-time MSP analysis as determined by 2.0% agarose gel electrophoresis is shown in A and B. C: Summary of *Ndrg2* methylation in 292 gastric cancers tissues, 292 para-cancerous histological normal tissues (PCHNTs) from the same patients and 88 non-cancer volunteers. Data shows the frequency of *Ndrg2* methylation (DNA methylation level ≥20%) in each group. D: 5-Aza-CdR or/and TSA treatments restored *Ndrg2* gene expression in *Ndrg2* silenced HGC-27 cells. Two gastric cancer cell lines (HGC-27 and SGC-7901) were treated with 1.0 μmol/L 5-Aza-CdR for 72 hours and/or 100 nM TSA for 24 hours. The methylated levels were determined by real-time MSP. We performed real-time RT-PCR analysis in triplicate for each cDNA sample and used median values in three experiments. The relative *Ndrg2* mRNA expression was normolized to the Gapdh of the same samples using the formula 2^−ΔΔCT^. The results were multiplied by 100 for a better visualization. The percentage of *Ndrg2* DNA methylation is shown on the left side; whereas the relative mRNA expression of *Ndrg2* is shown on the right side. E: Expression of *Ndrg2* at the protein level following 5-Aza-CdR and TSA treatments. Western blot analysis of HGC-27 cells following treatment with DMSO (control), 5-Aza-CdR, or 5-Aza-CdR/TSA for 72 hours demonstrate up-regulation of the Ndrg2 proteins in treated cells as compared to control (DMSO). β-actin is shown as a loading control.

RT-PCR analysis showed that *Ndrg2* mRNA expression is repressed in 4 of 5 gastric cancer cell lines examined and in human gastric cancer samples. To understand the molecular mechanism of the predominant transcriptional repression of *Ndrg2* in gastric cancer cells, we investigated the DNA methylation status of a CpG island encompassing the proximal promoter of *Ndrg2* using a real-time methylation-specific PCR (MSP). *Ndrg2* promoter hypermethylation were detected in HGC-27, AGS, MKN-45 and MKN-28 cell lines in which *Nrdg2* are silenced, but was not detected in SGC-7901 (Figure 2A). These results indicate that the transcriptional repression of *Ndrg2* in gastric cancer cells is highly correlated with promoter hypermethylation.

To confirm these results, we investigated the DNA methylation status in 292 paired tumor and PCHNT tissues by real-time MSP. The *Ndrg2* promoter were frequently hypermethylated in tumor tissues in contrast to the PCHNT tissues of the same individual. Some methyl CpG sites were also detected in PCHNT tissues but the frequency was much lower than that in tumor tissues (Figure 2B-C). According to our definition, we took 20% of methylation level as the threshold for DNA hypermethylation. Based on this criterion, the *Ndrg2* hypermethylation was detected in 65.8% (192/292) of GC tissues and 11.3% (33/292) of PCHNTs, respectively (*P*=0.000). The latter were found to be mostly partially methylation of *Ndrg2* gene, which methylation score less than 4. While the methylated frequency of *Ndrg2* in non-cancer controls were 4.0% (5/125). The frequency of *Ndrg2* methylation in GC tissues and PCHNTs was significant higher than that in non-cancer control (*P*=0.000, and *P*<0.05, respectively) (Figure 2C).

### Promoter methylation of *Ndrg2* gene was correlated with its down-regulation at both mRNA and protein levels

**Figure 3:**
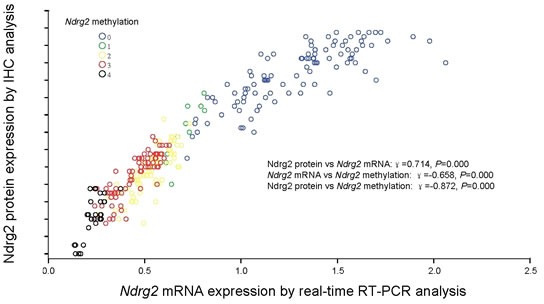
Correlation of *Ndrg2* methylation with Ndrg2 expression Correlation of *Ndrg2* methylation with *Ndrg2* mRNA level determined by real-time RT-PCR analysis and Ndrg2 protein expression determined by immunohistochemical analysis in 292 gastric cancer tissues. *Ndrg2* methylation scores inversely correlated with *Ndrg2* gene expression in both mRNA and protein levels.

To examine the relationship between *Ndrg2* methylation and *Ndrg2* expression, we compared the *Ndrg2* methylation level with *Ndrg2* mRNA or protein level determined by IHC using the Spearman correlation analysis. As shown in Figure 3, *Ndrg2* methylation significantly correlated with *Ndrg2* mRNA level (r=−0.658, *P*=0.000) and *Ndrg2* protein level (r=−0.872, *P*=0.000). And *Ndrg2* protein expression (determined by IHC analysis) in 292 GC tissues was closely correlated with *Ndrg2* mRNA level [lg(T/N)] (determined by RT-PCR) (r=0.714, *P*=0.000). To further confirm these results, two gastric cell lines (HGC-27 and SGC-7901) were treated with the de-methylating agent 5-aza-2′-deoxycytidine (5-Aza-CdR). As shown in Figure 2D, *Ngrg2* mRNA expression was reactivated in HGC-27 cells with significant demethylation of *Ndrg2* promoter by 5-Aza-CdR, indicating that *Ndrg2* is transcriptionally silenced in these cells by DNA hypermethylation. Interestingly, TSA treatment alone was effective in restoring the expression of *Ndrg2* gene in HGC-27 cells, but did not change the methylation levels, suggesting that histone modifications may also be involved in regulating the expression of *Ndrg2*. However, administration of TSA following 5-Aza-CdR had an additive effect in restoring gene expression and led to a further decrease in the methylation level of *Ndrg2* (Figure 2D). These results are in agreement with recent studies that suggested that TSA can have a demethylation effect in a gene-specific manner [29]. The western blot analysis using HGC-27 cell line as a model, confirmed the up-regulation of Ndrg2 proteins following the 5-Aza-CdR and 5-Aza/TSA treatments (Figure 2E). 5-Aza-CdR or TSA treatment alone or 5-Aza/TSA treatments did not have effect on the gene expression of *Ndrg2* at both mRNA and protein levels in SGC-7901 cells, in which the *Ndrg2* promoter was non-hypermethylated (Figure 2D-E). These findings indicate that promoter hypermethylation of *Ndrg2* is the major mechanism for silencing *Ndrg2* expression in GC.

Of note, the methylated status of *Ndrg2* significantly correlated with some clinicopathological parameters in GC: *H. pylori* (HP) infection (*P*=0.000), tumor size (*P*=0.001), venous invasion (*P*=0.000), lymphatic involvement (*P*=0.000), invasive depth (*P*=0.000), lymph node metastasis (*P*=0.000), distant metastasis (*P*=0.000) and clinical stage (*P*=0.000). However, there was no significant correlation between the methylated status of *Ndrg2* and gender, age at diagnosis, tumor site, histology and differentiation (Table 1).

### *Ndrg2* methylation is closely associated with the prognosis of the gastric cancer patients

**Figure 4:**
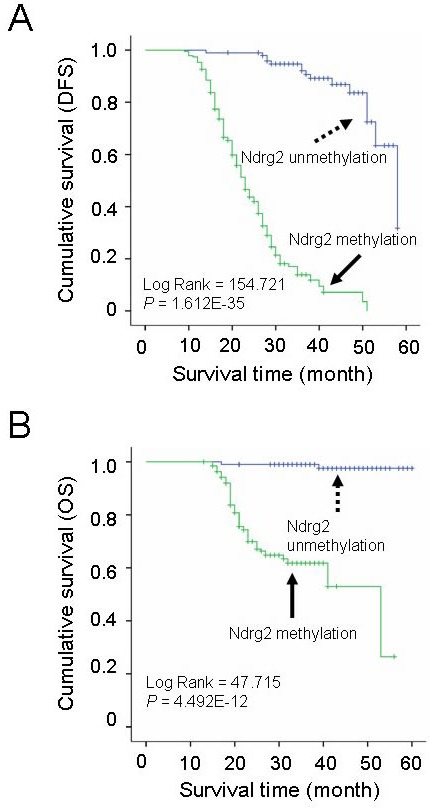
Correlation of Ndrg2 methylation level with survival of patients with gastric cancer A: Kaplan-Meier curves of overall disease-free survival (DFS) in patients with gastric cancer treated with primary gastrectomy according to the methylated level of *Ndrg2*. B: Kaplan-Meier curves of overall survival (OS) in patients with gastric cancer treated with primary gastrectomy according to the methylated level of *Ndrg2*.

We next investigated the association of *Ndrg2* methylation with patient survival. We analyzed both disease-free survival (DFS) and overall survival (OS). In both situations, *Ndrg2* methylation is significantly associated with poorer prognosis; shorter DFS and lower OS. The average durations of DFS and OS in patients with *Ndrg2* methylation in the tumors (Methylation score = 1-4) were significantly shorter than those of patients without *Ndrg2* methylation in the tumors (Figure 4). The average DFS in patients with *Ndrg2* methylation was 24.9 months vs 53.2 months in patients without *Ndrg2* methylation. The average OS in patients with *Ndrg2* methylation was 40.6 months vs 59.3 months in patients without *Ndrg2* methylation (Table 2). *Ndrg2* methylation was strongly associated with late stage tumors, but the association of *Ndrg2* methylation level with patient survival is independent on the tumor stage (Supplementary Figure 2). In addition to *Ndrg2* methylation, we observed that a number of other previously characterized clinical parameters were associated with patient survival (Table 2), including age at diagnosis, *H. pylori* infection, tumor size, differentiation, venous invasion, lymphatic invasion, invasive depth, nodal metastasis, distant metastasis, and TNM stage. In summary, these data indicate that in addition to a majority of clinical characteristics that have been shown to affect prognosis of gastric cancer patients, the methylation level of *Ndrg2* might serve as an independent marker to predict the DFS of the patients.

**Table 2:** Univariate and multivariate analysis of survival in 292 patients with gastric cancer according to clinicopathologic factors and *Ndrg2* methylation

Clinicopathologic factor	DFS							OS					
	Total	Survival (mo)	Univariate analysis		Multivariate analysis^§^			Survival (mo)	Univariate analysis		Multivariate analysis^§^		
	n		χ^2^	P-values	HR	(95% CI)	P		χ^2^	P-values	HR	(95% CI)	P
Gender													
Male	168	36.1	0.323	0.570	1.510	1.039-2.196	0.031	52.6	9.082	0.003	1.879	1.006-3.509	0.048
Female	124	35.0						45.9					
Age at diagnosis													
<60	109	39.5	6.627	0.010	0.948	0.659-1.365	0.774	53.2	9.359	0.002	1.054	0.513-2.165	0.886
≥60	183	33.7						47.8					
Tumor site													
Body/Antrum	56	35.8	0.081	0.776	0.982	0.654-1.473	0.929	49.4	2.310	0.129	1.771	0.810-3.870	0.152
Cardia	236	36.8						52.4					
Tissue type													
Diffuse	96	37.3	1.720	0.190	1.086	0.757-1.560	0.654	50.9	3.585	0.058	1.262	0.664-2.398	0.477
Intestinal	196	34.9						49.0					
H. *pylori*													
Negative	131	48.7	148.156	4.385E-34	2.613	1.549-4.409	3.185E-04	58.7	67.648	1.954E-16	4.695	1.191-18.513	0.027
Positive	161	24.3						38.5					
Tumor size													
<5cm	90	41.1	11.101	0.001	1.081	0.727-1.606	0.700	54.7	7.815	0.005	1.260	0.607-2.618	0.535
≥5cm	202	33.5						47.3					
Differentiation													
Poorly	101	34.3	4.214	0.040	0.896	0.609-1.318	0.576	47.4	6.050	0.014	0.674	0.335-1.356	0.269
Well/Medium	191	38.4						53.8					
Venous invasion													
No	140	46.3	95.980	1.160E-22	0.814	0.398-1.664	0.573	54.6	21.126	4.301E-06	0.574	0.196-1.682	0.312
Yes	152	25.7						43.3					
Lymphatic invasion													
No	116	48.1	93.128	4.903E-22	2.216	1.012-4.853	0.047	55.8	23.405	1.312E-06	1.444	0.387-5.393	0.585
Yes	176	26.8						44.1					
Invasive depth													
T1/T2	121	45.4	68.601	1.206E-16	1.615	1.082-2.411	0.019	55.6	23.521	1.236E-06	1.927	0.939-3.955	0.074
T3/T4	171	27.2						43.4					
Nodal metastasis													
No	104	47.8	65.994	4.523E-16	2.545	1.563-4.144	1.721E-04	53.2	5.589	0.018	1.814	0.952-3.455	0.070
Yes	188	28.5						46.2					
Distant metastasis													
No	220	41.4	256.750	8.769E-58	10.90	6.689-17.77	9.351E-22	57.9	309.243	3.192E-69	79.02	25.723-242.797	2.339
Yes	72	16.7			3	2		20.9			8	7	E-14
TNM stage													
I/II	84	54.6	140.758	1.817E-32	36.06	7.849-165.7	4.062E-06	59.6	40.712	1.764E-10	49468	1.224E-64-2.0	0.893
III/IV	208	24.9			4	04		34.9			.676	00E-73	
*Ndrg2* methylation													
MS^*^=0	100	53.2	154.721	1.612E-3	5.700	2.774-11.71	2.184E-06	59.3	47.715	4.929E-12	3.254	0.528-20.040	0.203
MS=1-4	192	24.9		5		6		40.6					

### *H. pylori* infection enhances DNMT activity and expression *in vitro*

**Figure 5:**
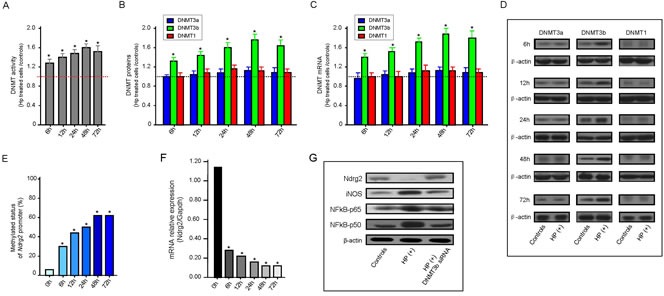
*H. pylori* infection enhances DNMT activity and NDMT3b expression *in vitro* Results from triplicate determination of total DNMT activity and individual DNMT protein assays are shown. Error bars represent S.E.M. A: Fold change of DNMT activity in HP-treated SGC-7901 cells corresponds to the equivalent amount of untreated control cells with a set value of 1.0 for each point in time. * *P* < 0.05 vs. untreated controls. B: The total DNMT activity was measured using the EpiQuik™ DNA Methyltransferase Activity/Inhibition Assay (Epigentek), and the individual DNMT proteins of interest (DNMT1, DNMT3a, or DNMT3b) was measured using the Epiquik DNMT1, -3a, and -3b assay kits, respectively. Fold change of DNMT protein level in HP-treated SGC-7901 cells corresponds to the equivalent amount of untreated control cells at each point in time using the set value 1.0. * *P* < 0.05 vs. untreated controls. C: Results were normalized to the Gapdh level of each sample and are expressed fold change of DNMT mRNA level in HP-treated SGC-7901 cells corresponds to the equivalent amount of untreated control cells at each point in time using the set value 1.0. * *P* < 0.05 vs. untreated controls. D: Western blot analysis of DNMT expression change in HP-treated SGC-7901 cells. Human β-actin is used as internal reference. E: Effects of HP infection on *Ndrg2* methylation in SGC-7901 cells at each point in time *in vitro*. * *P* < 0.05 vs. untreated controls (0 h). F: Effects of HP infection on *Ndrg2* mRNA expression in SGC-7901 cells at each point in time *in vitro*. *Ndrg2* mRNA level was determined by RT-PCR and adjusted for Gapdh. * *P* < 0.05 vs. untreated controls (0 h). G: Representative western blots of SGC-7901 cells treated with *H. pylori* or cells pretreated with *H. pylori* and then cocultured with DNTM3b siRNA were shown. Human β-actin is used as internal reference in each group of western blot analysis

After 6 h of *H. pylori* treatment, incubation of SGC-7901 cells led to a rapid and significant increase in total DNMT activity by 29.8% as compared to the controls. At later points in time, DNMT activity was stably increased by approximately 10% for 12h, 24 h, 48 h time point, and 5% for 72 h time point, respectively, where a total increase of more than 60% was observed (Figure 5A). No significant differences were detected for DNMT1 or DNMT3a protein levels between SGC-7901 cells with and those without *H. pylori* treatment at every time point (Figure 5B). The average DNMT3b protein level for the SGC-7901 cells with *H. pylori* treated was 1.3-fold at 6 h and reached a maximum of 1.8-fold at 48 h, compared to the controls (Figure 5B).

Expression of *DNMT1*, *DNMT3a* and *DNMT3b* were then investigated by quantitative real-time RT-PCR. *H. pylori* treatment significantly increased mRNA for *DNMT3b* while no changes were observed in *DNMT1* and *DNMT3a* levels (Figure 5C). These findings were corroborated by western blot analysis showing a strong increase of DNMT3b protein in *H. pylori* treatment cells but not of DNMT1 and DNMT3a (Figure 5D). Here, a transient increase in DNMT3b protein levels was observed after 6 to 72 h in *H. pylori* treatment cells. The DNMT3b expression at both mRNA and protein levels were rapidly increased by *H. pylori* treatment for only 6 h, and increased continuously until 72 h, however, this trend was tending towards stability.

### Effects of *H. pylori* treatment on *Ndrg2* methylation and its molecular mechanism underlying this regulation *in vitro*

We next investigated whether the increase of DNMT activity and expression is also reflected on the change of methylated status of *Ndrg2* promoter. Real-time MSP was performed for *Ndrg2* in SGC-7901 cells treated by *H. pylori* at the given time points. The increase of *Ndrg2* methylation (3.8-fold) and the decrease of *Ndrg2* mRNA (4.1-fold) in SGC-7901 cells were observed after *H. pylori* treatment only 6 h when compared to the level of untreated controls. *Ndrg2* methylation level was increased as much as approximately 7.8-fold, while *Ndrg2* mRNA expression was decreased as much as approximately 8.5-fold at 48 h time point in the treated cells as compared to the controls (Figure 5E, F).

**Figure 6:**
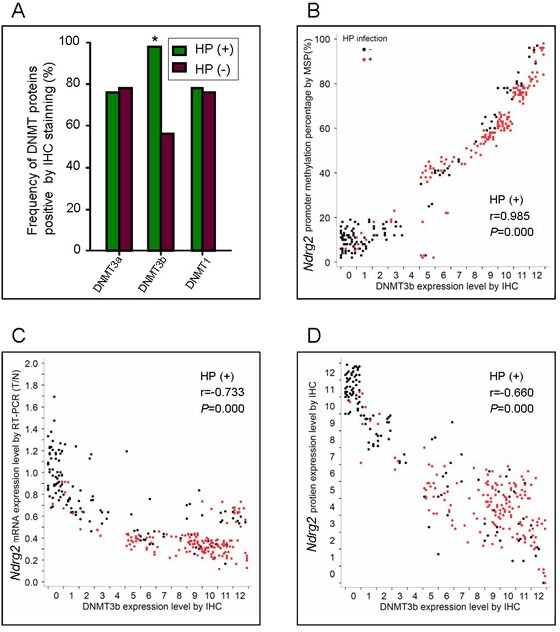
Correlation between DNMT3b expression and *Ndrg2* methylation or Ndrg2 expression in primary gastric cancer tissues A: Comparison of frequency of DNMT proteins expression between in GC with HP infection and those without HP infection, IHS, immunohistochemical score. B: Correlation of DNMT3b protein level with *Ndrg2* methylation in GC tissues. C: Correlation of DNMT3b protein level with *Ndrg2* mRNA expression in GC tissues. D: Correlation of DNMT3b protein level with Ndrg2 protein expression in GC tissues.

NFκB plays a critical role during inflammatory response to *H. pylori* infection. We next examined the protein expression of iNOS, NFκB-p65, and NFκB-p50 in the SGC-7901 cells. As shown in Figure 6, in *H. pylori*-treated cells, there was a great increase in iNOS, NFκB-p65, and NFκB-p50 protein expression (2-fold to 5-fold), whereas the expression of Ndrg2 protein was down-regulated (approximately 2-fold to 3-fold), corresponds to the equivalent amount of untreated control cells. Again, when the *H. pylori*-pretreated cells were cocultured with DNMT3b siRNA, the effects were diminished, and protein expression returned to levels comparable to control cells (Figure 5G). These findings were similar in degree to the mRNA expression analysis of *Ndrg2*, *iNOS, NFκB-p65*, and *NFκB-p50* (data no shown). Promoter methylation of *Ndrg2* was induced in cells SGC-7901 treated with *H. pylori* (Figure 5E). No methylation alleles were observed in the control cells without *H. pylori* challenge. Similarly, the promoter methylation of *Ndrg2* gene disappeared when SGC-7901 cells were pretreated with *H. pylori* and then were cocultured with DNMT3b siRNA (data not shown).

### Correlation between DNMT3b expression and *Ndrg2* methylation or *Ndrg2* expression in primary gastric cancer tissues

To investigates the varied expression of DNA methyltransferase (DNMT) proteins in gastric cancer and their relationship with *Ndrg2* methylation or *Ndrg2* expression. Immunohistochemistry was used to detect the expression of the 3 DNMTs in GC tissues. We discovered that the positive rates of DNMT1, DNMT3a, and DNMT3b expression in GC tissues were 78.4% (229/292), 78.4% (229/292), and 79.8% (233/292), respectively, and they were significantly higher than those of three PCHNTs (19.2%, 18.2%, and 14.4%) and non-cancer control tissues (8.8%, 7.2%, and 6.4%). DNMT1 was well distributed in the cytoplasm and nuclei of tumor cells or glands, while DNMT3a and 3b were well distributed only in the cytoplasm, as shown by staining a dark brown color. A significant correlation between the DNMT1 and DNMT3a proteins (*P*<0.01), a low correlation between DNMT3a and DNMT3b (*P*=0.06), and no correlation between DNMT1 and DNMT3b (*P*>0.05) were found.

DNMT3b protein level significantly correlated with *H. pylori* (HP) infection in GC tissues (*P*=0.000). The frequency of DNMT3b expression in GC tissues with HP infection (98.8%, 159/161) was significant higher than that those without HP infection (56%, 74/131) (*P*<0.05, Figure 6A). However, there was no significant correlation between DNMT1 or DNMT3a protein level and HP infection (both *P*>0.05). The DNMT1 or DNMT3a protein in GC tissues with HP infection and those without HP infection were also compared. They coincide well, which the frequency of DNMT3a was 78.3% (126/161) in HP(+) and 78.6% (103/131) in HP(−) groups, DNMT1 was 78.9% (127/161) in HP(+) and 77.9% (102/131) in HP(−) groups (Figure 6A).

The expression of DNMT3b protein in GC tissues significantly correlated with the level of *Ndrg2* methylation (r=0.985, *P*=0.000), the level of *Ndrg2* mRNA (r=−0.733, *P*=0.000) and the level of Ndrg2 protein (r=−0.660, *P*=0.000), respectively. (Figure 6B, C and D). These findings were consistent with that detected in SGC-7901 cells *in vitro*.

## DISCUSSION

In present study, we investigated the biological function and clinical significance *Ndrg2* in gastric cancer progression. We found that *Ndrg2* was frequently down-regulated in gastric cancer and provided evidence suggesting that this observation can have significant implications in the negative regulation of gastric cancer progression. Present studies suggest that *Ndrg2* might be a novel candidate suppressor of tumor progression in gastric cancer. First, down-regulation of *Ndrg2* expression is frequently observed in primary tumors and tumor cell lines. Our data showed that *Ndrg2* expression was significantly reduced in the majority of gastric cancers compared with adjacent non-tumor tissues, which is similar to others [27, 28]. Immunohistochemical analysis of Ndrg2 protein expression in gastric cancer indicated that Ndrg2 expression loss is closely correlated with tumor size, histological differentiation, venous invasion, lymphatic involvement, invasive depth, lymph node metastasis and clinical stage (all *P*<0.05) (Table 1). We also found that *Ndrg2* down-expression is one of the affecting factors on tumor progression by multivariate analysis. Together, these data indicate that *Ndrg2* might have a clinical significance as a marker associated with negative regulation of tumor progression in gastric cancer.

The loss of *Ndrg2* expression could have been caused by a number of genetic or epigenetic events during gastric cancer progression. In the present study, we showed that the CpG sites in the *Ndrg2* promoter were mostly hypermethylated in gastric cancer cell lines and primary tumors, whereas those in normal gastric tissues remained unmethylated. Loss of *Ndrg2* expression due to promoter hypermethylation has been reported in some studies [17-28]. We also found that the loss of *Ndrg2* expression in primary gastric cancer is carried on to the secondary tumors formed during metastatic progression, likely due to inheritance of the promoter hypermethylation pattern at *Ndrg2* locus (data no shown). Therefore, we conclude that methylation-induced promoter inactivation might be the major cause of the frequent loss of *Ndrg2* expression in gastric cancer. Using two *in vitro* cells model of SGC-7901 and HGC-27, we confirmed that DNA methylation is the mechanism underlying *Ndrg2* gene silencing. 5-Aza-CdR treatment significantly restored *Ndrg2* expression in *Ndrg2* silenced gastric cancer cell line HGC-27 cells. These data clearly proved that frequent *Ndrg2* down-expression in gastric cancers is regulated (at least in part) by *Ndrg2* promoter hypermethylation. More importantly, we found that both *Ndrg2* hypermethylation and down-regulation of *Ndrg2* were significantly correlated with several important clinical parameters such as lymph node metastasis, T-stage, clinical stage, etc (all *P*<0.05), suggesting that frequent dysfunction of *Ndrg2* through its promoter methylation play crucial roles in malignant progression of human gastric cancer and may have an important impact on the metastasis and poor survival of gastric cancer patients [27, 30]. Recently, it has been indicated that a lack of *Ndrg2* is associated with oncogenic properties through the loss of its role as a tumor suppressor, and that *Ndrg2* is an independent poor prognostic factor predicting survival in clear cell renal cell carcinoma, suggesting that it can serve as a novel prognostic biomarker [31].

Just as important, we found that *Ndrg2* expression loss via DNA methylation is closely correlated with H. *pylori* infection (*P=*0.000) (Table 1). *H. pylori* is known to participate in gastric carcinogenesis. Chronic inflammation of the gastric epithelium due to *H. pylori* infection that may lead to an increased risk of gastric cancer [32-34]. In gastric carcinogenesis, aberrant promoter methylation plays a major role by inactivating tumor-suppressor genes. *H. pylori* may contribute to carcinogenesis through the induction of aberrant methylation in gastric epithelial cells, although further study is necessary to elucidate the detailed molecular mechanisms underlying the induction of aberrant promoter methylation in response to *H. pylori* infection [35-39].

Here, we analyzed *H. pylori* infection and aberrant *Ndrg2* methylation in gastric cancer, the association between *H. pylori* infection and *Ndrg2* methylation and its possible mechanism. Our results suggest that the mechanism that accounts for *Ndrg2* methylation in *H. pylori*-related gastric cancer is elevated DNMT activity secondary to the up-regulation of DNMT3b. DNMT3b protein is significantly elevated in HP-related GC tissues and SGC-7901 cells with HP treatment. These SGC-7901 cells treated by HP exhibit aberrantly increased DNMT activity and DNMT3b expression, correspondingly increase of *Ndrg2* methylation compared to both those GC tissues and SGC-7901 cells counterparts without HP infection. These results are in agreement with those of other recent studies, in which aberrant DNMT3b up-regulation was implicated in the methylation abnormalities of tumors [40-42]. The mechanisms by which *H. pylori* might promote carcinogenesis are not well understood. *H. pylori* infection, a major risk factor for gastric cancer, induces the methylation of specific genes in the gastric mucosa [35-39]. Accumulating findings support the idea that the inflammatory response triggered by *H. pylori* infection is responsible for inducing altered DNA methylation [43]. This suggests that *H. pylori* might interact with the CpG island methylator mechanism of carcinogenesis in response to inflammation. The hypermethylation of CpG islands, and concomitant loss of gene expression, is the best characterized epigenetic change to occur in tumors and is catalyzed by DNMT1, DNMT3a, and DNMT3b [44, 45]. Classically, DNMT1 is considered a maintenance methyltransferase whereas DNMT3b may be more important for de novo CpG methylation, although this distinction is not absolute [46, 47]. DNMT3b up-regulation has been observed in gastric cancer [48, 49], but whether *H. pylori* infection during gastric cancer progression have any role in regulating its expression is unclear. Our results suggest that *H. pylori*, as an initiator of the inflammatory tumor microenvironment, might promote the tumorigenesis and progression of gastric cancer through the regulation of epigenetic tumor suppressor genes silencing such as *Ndrg2* methylation. We also found that *H. pylori* infection induced up-regulation of the DNMT3b but not the DNMT3a and DNMT1 *in vitro* model.

The potential molecular mechanism underlying the regulation of *Ndrg2* methylation by *H. pylori* infection indicates the role of *H. pylori* in epigenetic modification through an activation of NF-κB pathway that links inflammation to carcinogenesis. Chronic inflammation is known to promote certain types of cancers, such as gastric cancers. Chronic inflammation and aberrant methylation including promoter and histone methylation are frequently found in *H. pylori*-associated gastric diseases [50, 51]. Liang et al reported that PI3K/AKT-Sp1-RBP2-Cyclin D1 pathway may serve as a novel mechanism for gastric epithelial cell malignant transformation and then gastric cancer (GC) triggered by CagA (+) *H. pylori* [51]. Hence, chronic inflammation caused by *H. pylori* infection is suggested to be an inducer of aberrant methylation [52, 53]. Our findings were further supported by some recent studies showing that gastric cancer is triggered by *H. pylori* infection in induction of aberrant DNA methylation [50, 52, 53]. However, it is necessary that given a thorough *in vivo* study using animal models to further characterize the mechanistic role of NF-κB pathway in the interaction between H.*pylori*-induced host inflammatory response and DNA methylation. Even so, we also recognize the diversity and complexity of *H. pylori*-mediated target genes change. The NDRG protein family consists of 4 members, *Ndrg1*, *Ndrg2*, *Ndrg3* and *Ndrg4*, which share 57-65% amino acid identity [10]. We recently also found that there is different effect of *H. pylori* infection on other members of Ndrg family. Increase of *Ndrg4* promoter methylation triggered by *H. pylori* was found, which is similar to *Ndrg2*. However, up-regulation of *Ndrg1* and *Ndrg3* was observed when *H. pylori* infection (data no shown). Recently, Bhardwaj et al [54] found that regulation of p53 and E-cadherin is tightly linked through the p53 stress response mechanism that is inhibited by *H. pylori* via activation of Erk1/2-HDM2-p53 pathway leading to survival of damaged cells. Zaika et al [55] suggest that *H. pylori* has developed clever mechanisms to counteract the function of p53 during the process of evolutionary adaptation to the host environment, and such alteration may increase the risk of tumor development. Zhou et al [56] carried out the study on the molecular mechanism of miRNAs mediated by *H. pylori* in gastric cancer, indicated that miR-203 functions as a growth-suppressive miRNA in *H. pylori* related GC, and that its suppressive effects are mediated mainly by repressing CASK expression. All these findings reflect the complexity of *H. pylori* infection as the strongest known risk factor for gastric cancer.

In summary, we reported the frequent loss of *Ndrg2* expression in gastric cancer caused by promoter CpG sites hypermethylation, the significance of the relationship between *Ndrg2* expression loss and tumor progression in gastric cancer patients, and the induction of *Ndrg2* methylation by *H. pylori* infection via the activation of NFκB pathway and up-regulation of DNMT3b. Our results provided the evidence that *Ndrg2* could contribute to the negative regulation of gastric cancer progression. Further investigations on the molecular mechanism of *Ndrg2* methylation in gastric cancer mediated by *H. pylori* infection may offer a novel approach for the prevention and treatment of gastric cancer. Understanding these mechanisms could clarify the process of gastric carcinogenesis and progression, and application of this knowledge for clinical use could aid in diagnosis, risk management, prevention and molecular target therapy.

## MATERIALS AND METHODS

### Cell lines and *H. pylori* strain

The human gastric cancer cell lines (AGS, SGC-7901, HGC-27, MKN45 and MKN-28) were grown in RPMI-1640 medium (GIBCO, Rockville, MD), supplemented with 10% fetal bovine serum (FBS), 100 U/mL penicillin, and 100 U/mL streptomycin at 37°C and 5% CO_2,_ respectively. 5-aza-2′-deoxycytidine (5-Aza-CdR) and trichostatin A (TSA) (Sigma-Aldrich, USA) were dissolved in dimethyl sulfoxide (DMSO). Cultured cells were seeded at a density of 5 × 10^5^ cells per flask. The cells were in logarithmic phase and the number of viable cells was 95% to 100% before the addition of 5-Aza-CdR to the culture medium at a final concentration of 1.0 μmol/L. The medium was changed daily and the drug concentration was maintained. The cells were collected after 72 h of drug treatment. Cells were exposed to TSA at a final concentration of 20 ng/ml for 24 hours. Cells in the untreated group were cultured in normal complete culture medium for 72 h.

*H. pylori* strain NCTC 11637 was maintained on brain heart infusion agar medium (OXID, Basingstoke, UK) containing 5% sheep blood incubated at 37^o^C in 5% O^2^ for a minimum of two and a maximum of four passages from frozen stocks. *H. pylori* bacteria were added to cultured SGC-7901 cells at ratio of 1:10 and cocultured till total RNA was extracted at 6, 12, 24, 48, and 72 h after *H. pylori* induction.

### Patients and tissue samples

A total of 292 patients with gastric cancer, who underwent curative surgery without prior treatments at Zhejiang Province Cancer Hospital from January 2008 to December 2010, were enrolled in this study. Patients with other gastric tumors, such as neuroendocrine tumors, lymphoma, and sarcoma, were excluded from this study. The patients' medical records were reviewed to obtain data including age at diagnosis, sex, tumor location, tumor size (diameter), nerve invasion, and American Joint Committee on Cancer stage. The mean age of patients at tumor resection was 56.1 years; 168 (57.5%) were male and 124 (42.5%) were female. Demographic, clinical and histopathological parameters of these cases were shown in Table 1. Tumor specimens and corresponding para-cancerous histological normal tissues (PCHNTs) were collected at the time of surgery. Paired PCHNTs were obtained from a 5-cm distance from the tumor edge and was assessed microscopically for the presence of normal cells and absence of dysplastic cells. The use of the tissue specimens for the present study obtained patient informed consent, and the use of the human specimens was approved by the Zhejiang Province Cancer Hospital Institutional Review Board. As a measure of prognosis, we analyzed the clinical data concerning disease-free survival (DFS) and overall survival (OS), defined as the time from surgery data to first recurrence (DFS), or death (OS) by gastric cancer. All recruited patients had been followed-up periodically until the due date. The mean follow-up duration was 28 months, ranging from 11 to 59 months.

Antral mucosa biopsy specimens from 125 non-cancer volunteers by gastroscopy were randomly collected as controls within the same period, including 74 men and 51 women, with an average age of 51.8 years old. Among these volunteers, 37 patients were diagnosed with dysplasia (DYS), 48 patients were diagnosed with chronic non-atrophic gastritis (CAG) and 40 healthy individuals without other digestive system diseases. The 125 non-cancer volunteers provided written informed consent. Part of the specimens were from Zhejiang Province People's Hospital, the First People's Hospital of ChunAn County and the Center for clinical laboratory of DiAn. The use of the specimen for this study was approved by the Institutional Review Board of Medical Ethics of above institutes.

### Real-time RT-PCR analysis

The mRNA expression of *Ndrg2* was analyzed by real-time RT-PCR. Total cellular RNAs were extracted using the Trizol (Gibco) one-step method. A total of 3 μg total RNA was subjected to reverse transcription using M-MLV reverse transcriptase (Promega). The glyceraldehyde phosphate dehydrogenase (*Gapdh*) was selected as the internal reference. Real-time RT-PCR was performed using the following primers: *Ndrg2* (F)-5′-atc tct gga cca gct tgc ag-3′, *Ndrg2* (R)-5′-tat ctc gcc agg atg tag gc-3′; *Gapdh* (F)-5′-ctg ggc tac act gag cac c-3′, *Gapdh* (R)-5′-aag tgg tcg ttg agg gca atg-3′. Primers for *DNMT1*, *DNMT3a* and *DNMT3b* were purchased from Qiagen. The 2^−ΔΔCt^ method was used to calculate relative changes in gene expression determined from real-time RT-PCR experiments.

### Immunohistochemical analysis

Immunohistochemistry was performed as described previously [57]. Avidin–biotin–peroxidase method was used for immunohistochemical assay. All sections were deparaffinized with xylene and dehydrated with gradient alcohol followed by inactivation of endogenous peroxidase activity by 0.5% H_2_O_2_ in methanol for 10 mins. Non-specific binding was blocked by incubation with 10% normal goat serum in phosphate-buffered saline (PBS) for 1 h at room temperature. Then, slides were incubated with primary antibodies: anti-Ndrg2 (Abnova, Walnut, CA, USA), and anti-DNMT1/3a/3b (Santa Cruz Biotechnology, USA), respectively, at 4°C overnight, followed by biotinylated goat anti-mouse IgG (1:400; Sigma, St. Louis, MO, USA) or goat anti-rabbit IgG (1:400; Sigma) for 1 h at room temperature. Then streptavidin–biotin–peroxidase- complex assay was performed. A brown color was indicative of protein positive expression. Peroxidase activity was developed by incubating with 0.1% 3,3-diaminobenzidine (Sigma) in PBS with 0.05% H_2_O_2_ for 5 mins at room temperature. The IHC staining score is determined by three independent pathologists based on combining staining frequency and intensity as previous described [50].

### Western blot analysis

Thirty specimens of gastric cancer and their paired normal gastric epithelium tissues from 292 gastric cancer cases were prepared for western blot analysis. Total protein was extracted and then quantified using the Lowry method [58]. Western blot analysis was performed as previous described [57]. β-actin (TaKaRa Co. Ltd, Kusachi, Japan) was served as an internal control. The bands density was measured by the chemiDoc-XPS Gel imaging system with Epson color image scanner and Image-Pro Plus software and normalized to the β-actin. The experiments were repeated three times and the mean value was calculated for statistical analysis.

### DNA extraction, bisulfite modification and real-time methylation-specific PCR

Serial 5-μm-thick sections that contained carcinoma and non-neoplastic tissues were mounted on non-coated glass slides and dried at 37°C overnight. After deparaffinization and staining with Hematoxylin and Eosin (HE), we collected 5000 nuclei from 5 to 10 serial sections using a 27G needle. The collected target cells were treated with 40 μl of 200 μg/ml proteinase K (Sigma-Aldrich) at 42°C, for 72 hours. DNAs were modified by sodium bisulfite using the EpiTect Bisulfite kit (Qiagen Inc.) following manufactory's instructions. Modified DNAs were analyzed by real-time methylation-specific PCR (MSP) on a ABI7500 PCR (ABI Co.) using the SYBR Premix Taq ExTaq Kit (TaKaRa Co. Ltd). The specific primers for detection of *Ndrg2* CpG island methylation and unmethylation were designed according to previous report [59]. The percentage of methylated DNAs in the samples were calculated according to the Ct value and a standard curve, and methylated DNA was scored as previous described [39, 57]. All samples were analyzed with primer sets for both methylated and unmethylated DNA. The relative amount of methylation in each unknown sample was calculated as the percentage methylation=100 × (number of copies of methylated DNA / [number of copies of methylated + unmethylated DNA]) [60]. The sum of unmethylated plus methylated DNA (U + M) was used as an approximation of the total number of target gene copies. Methylated DNA was scored according to the methylated percentage (0, <20%; 1, 20%-40%; 2, 40%-60%; 3, 60%-80%; and 4, >80%; scores of 0, 1-3, and 4 were considered unmethylated, partially methylated, and fully methylated, respectively) [39, 57, 60]. The cut off threshold for DNA hypermethylation was set as 20% based on control normal samples and internal quality controls provided in the real-time MSP analysis.

### Measurement of DNA methyltransferase (DNMT) activity

Nuclear protein was isolated with EpiQuik™ Nuclear Extraction Kit I (Epigentek, Brooklyn, NY, USA) from SGC-7901 cells exposed to *H. pylori* or from untreated control cells. After protein quantification with Total Protein Kit (Micro Lowry, Peterson's Modification; Sigma-Aldrich Chemie GmbH, Munich, Germany), 12 μg of nuclear protein was used to measure total DNMT activity with the EpiQuik™ DNA Methyltransferase Activity/Inhibition Assay (Epigentek) in accordance with the manufacturer's instructions. Nuclear extracts were assayed for individual DNMT proteins of interest (DNMT1, DNMT3a, or DNMT3b) using the Epiquik DNMT1, -3a, and -3b assay kits, respectively (Epigentek, Brooklyn, NY, USA), according to manufacturer's protocol.

### Knocking down of DNMT3b with RNA interference

Sequences of RNA interference (RNAi) oligonucleotides were as follows: non-silencing small interfering RNA (siRNA), UUCUCCGAACGUGUCACGU; DNMT3b siRNA, GATCAAGCTCGCGACTCTC (GeneChem Company, Shanghai, China). Each RNAi oligonucleotide was transfected into cells using the Lipofectamine 2000 transfection kit (Invitrogen, Carlsbad, CA) for 72 h. Cells were harvested and proteins were extracted for western blotting analysis.

### Statistical analysis

SPSS 17.0 statistical software was adopted for data analysis. Counting data comparisons between groups were subjected to the χ^2^ test or Fisher's exact test. Survival analysis was computed by means of the Kaplan-Meier method and significance were assessed by means of the log-rank test. A univariate analysis of the Cox regression model was used to determine prognostic factors, and multivariate analysis with the Cox regression model was used to explore combined effects. Pearson correlation analysis was used to analyze the correlation between *Ndrg2* methylation and DNMT1/3a/3b protein. We examined the direct effects of sex, age, tissue type, *H. pylori*, tissue size, differentiation, T stage, N stage, and *Ndrg2* methylaion in COX models. For all statistical analyses, *P* values <0.05 were considered to be statistical significance.

## SUPPLEMENTARY MATERIAL AND FIGURES


