# Supplementary material for: Ndrg2 promoter hypermethylation triggered by helicobacter pylori infection correlates with poor patients survival in human gastric carcinoma
Source: Oncotarget. 2015 Mar 15;6(10):8210–25. doi: 10.18632/oncotarget.3601 (PMC4480746; doi:10.18632/oncotarget.3601)
Supplement: Supplementary file 1 [file oncotarget-06-8210-s001.pdf]

Ndrg2 promoter hypermethylation triggered by helicobacter pylori infection correlates with poor patients survival in human gastric carcinoma

Supplementary Material

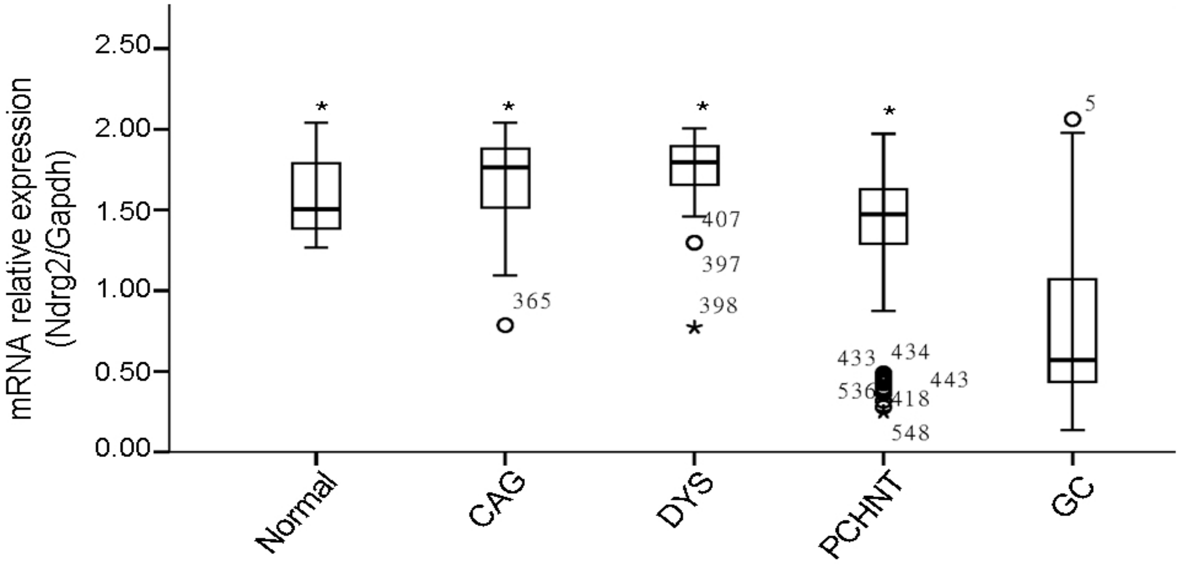

Supplementary Figure 1: **Expression of *Ndrg2* mRNA is robustly reduced in primary gastric carcinomas**.

Ndrg2 mRNA expression level in Normal, CAG, DYS, PCHNT and GC tissues were determined by real-time RT-PCR and was normalized to *Gapdh*. Normal: healthy individuals; CAG: chronic non-atrophic gastritis; DYS: dysplasia; PCHNT: para-cancerous histological normal tissue; GC: gastric cancer.

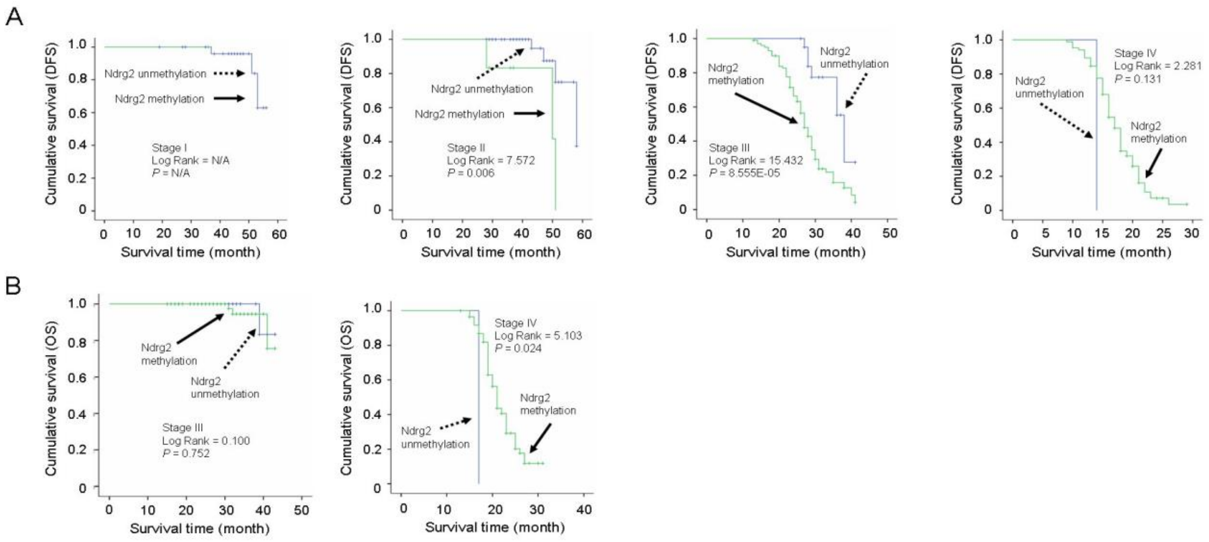

Supplementary Figure 2: **Correlation of *Ndrg2* methylation level with survival of GC patients based on different TNM stages.**

A: Kaplan-Meier curves of disease-free survival (DFS) in patients with gastric cancer treated with primary gastrectomy according to the methylated level of *Ndrg2*, and its stratifications based on different TNM stages.

B: Kaplan-Meier curves of overall survival (OS) in patients with gastric cancer treated with primary gastrectomy according to the methylated level of *Ndrg2*, and its stratifications based on different TNM stages.
